# Csf1^+^ AD-MSCs promote stroke repair by activating the resident microglia

**DOI:** 10.3389/ebm.2025.10611

**Published:** 2025-08-20

**Authors:** Jiguang Hou, Sunfu Zhang, Shuang Luo, Xiao Zuo, Fei Ma, Huizhen Wang, Pengfei Han, Ping Zhu, Ning Wang, Xiaoming Hou, Jin Li

**Affiliations:** ^1^ Department of Neurosurgery, West China Hospital of Sichuan University, Chengdu, China; ^2^ Department of Neurosurgery, Third People’s Hospital of Chengdu, Chengdu, China; ^3^ Department of Neurosurgery, Fifth People’s Hospital of Chengdu, Chengdu, China; ^4^ Tasly Stem Cell Biology Laboratory, Tasly Group, Tianjin, China; ^5^ Regenerative Medicine Research Center, West China Hospital of Sichuan University, Chengdu, China

**Keywords:** stroke, MSCs, microglia, single-cell transcriptome sequencing, subgroup

## Abstract

The potential of mesenchymal stromal cells (MSCs) in the treatment of hemorrhagic stroke has been demonstrated; however, their clinical efficacy remains inconsistent and further comprehensive studies on their mechanism of action are warranted. In this study, the intracerebral hemorrhage (ICH) rat model was used for intravenous infusion of adipose-derived mesenchymal stromal cells (AD-MSCs) 24 h after modeling. Histopathological techniques and single cell transcriptome sequencing techniques were used to study the mechanism of AD-MSCs promoting the repair of damaged brain tissue. The results indicated that AD-MSCs markedly promote the repair of damaged brain tissues and restored neural function. Single-cell transcriptome sequencing further revealed that this therapeutic effect is specifically through the inhibition of monocyte infiltration in injured brain tissue, promotion of resident microglia proliferation and signaling pathways linked to immune response and neuroprotection. These processes are closely tied to the Csf1^+^ subgroup of AD-MSCs. For acute hemorrhagic stroke, Csf1^+^ AD-MSCs promote the repair of damaged brain tissue by activating resident microglia and inhibiting monocyte infiltration. This study offers novel insights into the mechanisms underlying MSC-based stroke treatment and supports the potential for stable and efficacious MSC therapies.

## Impact statement

This study uncovers a critical mechanism by which adipose-derived mesenchymal stromal cells (AD-MSCs) promote brain repair in hemorrhagic stroke, revealing that the Csf1^+^ AD-MSCs subgroup enhances neuroprotection by activating resident microglia while suppressing monocyte infiltration. By integrating single-cell transcriptomics with functional validation, we resolve longstanding inconsistencies in MSC therapeutic efficacy and identify cellular heterogeneity as a key determinant of treatment outcomes. These findings provide a roadmap for optimizing MSC-based therapies through targeted enrichment of functional subpopulations, paving the way for standardized, high-efficacy treatments in stroke and potentially other neuroinflammatory disorders. This work bridges mechanistic insights with clinical translation, offering a transformative strategy to improve regenerative medicine for neurological injuries.

## Introduction

Hemorrhagic stroke is a common subtype of stroke, characterized by high rates of disability and mortality [1, 2]. With the development of cell therapy technology, MSCs have shown potential to promote the repair of injured nerve tissue. Animal studies haves shown that MSCs promote nerve regeneration, restore motor function, and hold promise for stroke treatment [3, 4]. However, clinical trials have identified a significant challenge–the inconsistent therapeutic effects of MSCs, limiting their widespread use [5–7]. More efforts are needed to elucidate the therapeutic mechanisms of MSCs.

Numerous studies have reported that MSCs exert therapeutic effects by participating in immune regulation at the site of injury [8, 9]. Microglia, as resident immune cells in brain tissue, play a crucial role in the repair of neural tissue damage. Under normal physiological conditions, microglia remain in a quiescent state, contributing significantly to maintaining brain tissue homeostasis [10, 11]. Following injury, resident microglia become activated and recruit bone marrow-derived inflammatory cells to infiltrate the area, resulting in the production of substantial inflammatory factors that cause severe damage to neural cells [12]. A pile of evidence demonstrated the immunomodulatory effects of MSCs on microglia in stroke model [13–15]. Results based on a model of traumatic brain injury in rats showed that intraventricular injection of MSCs 24 h after injury significantly inhibited the activity of microglia and promoted injury repair [16]. However, other studies have found that intravenous MSCs infusion 2 days after middle cerebral artery occlusion (MCAO) modeling does not suppress immune response, but aggravate inflammation and brain tissue damage, manifested by the increase of microglia and increased secretion of inflammatory factors such as TNF-α and IL-1β [17, 18]. These paradoxical phenomena suggest that the 24 h after injury may be the key time window for MSCs to play the role of immune regulation and promote the repair of damaged tissues.

Additionally, a considerable amount of research has shown that MSCs promote the transformation of microglia from the M1 type to the M2 type, promoting the repair of damaged brain tissue [19, 20]. Although the activation of M1 microglia is generally considered to be associated with the occurrence of inflammation and neurological injury, however, studies suggest that M1 microglia is necessary for clearing necrotic tissue debris and promoting tissue repair in the early stages of injury. Research by David et al. demonstrated that depleting microglia cell in brain tissue before inducing injury exacerbates the damage [21]. Studies based on single-cell RNA sequencing technology have revealed that during the repair of neural tissue injury, microglia cells exhibit dynamically changing and diverse cell phenotypes that cannot be simply categorized as M1 or M2 [22, 23]. Consequently, the mechanism of MSCs in promoting the repair of injured neural tissue cannot be simply defined as immune suppression; rather, it involves a complex interplay of immune modulation and tissue repair processes.

In this study, we constructed ICH experimental animal models utilizing Sprague-Dawley (SD) rats. To more accurately analyze the role of MSCs in damaged brain tissue, allogeneic AD-MSCs were administered via intravenous injection 24 h post-model establishment. By integrating histopathological techniques with single-cell transcriptome sequencing, we systematically analyzed the impact of AD-MSCs on brain microglial and the repair mechanisms of injured brain tissue. Furthermore, we elucidated the underlying molecular mechanisms. The findings from this research will offer a significant foundation for future clinical treatments.

## Materials and methods

### Animals

All animal procedures described here were approved by the Institutional Animal Care and Use Committee at Sichuan University West China Hospital.

The work has been reported in line with the ARRIVE guidelines 2.0.

### Hemorrhagic stroke model in rats

#### Experimental animals

Male rats were exclusively used in this study to preclude the neuroprotective effects of estrogen on brain injury and to circumvent operational variability associated with the estrous cycle in females. This approach eliminates confounding hormonal protection (e.g., reduced hematoma volume, accelerated recovery) and ensures model reproducibility by avoiding fluctuations in blood-brain barrier permeability and coagulation function inherent to the female estrous cycle [24–26]. The experimental animals were healthy male SD rats, 8–10 weeks old, weighing 220–280 g.

#### Modeling methods

The ICH model of the right striatum of rats was established by using the collagenase-induced ICH model reported by Rosenberg et al.

#### Anesthesia of animals

The rats were first induced with isoflurane at 4% concentration, the head surgical site was shaved and the surgical area was disinfected with iodophor, then the rats were fixed on a stereoscope, and the anesthesia was maintained with isoflurane at 2% ± 1% concentration for modeling surgery.

#### Modeling operation procedure

The skin was cut 1.0 cm at the median sagittal position of the head, and the periosteum was cut to the skin to reveal. Then a sterile cotton swab dipped in 3% hydrogen peroxide was used to corrode the outer mold of the skull, exposing the coronal suture and the fontanel. According to the brain atlas, the position of the right striatum was 3 mm beside the bregma, 0.2 mm behind it, and 5.6 mm deep. At the location, drill a small round hole with a dental drill to reach the surface of the hard membrane. Type Ⅶ collagenase was extracted with a microsyringe of 0.5 U/μL, and the microsyringe was fixed on the stereoscope, the needle was vertically inserted into the subdural 5.6 mm along the drilling hole, and the collagenase was slowly pushed into the brain for about 2 min, and the needle was slowly withdrawn to the extracranial for about 2 min after the needle was stopped. Finally, the bone holes were closed with bone wax, and the scalp was sutured and disinfected with iodophor.

#### Model identification and enrollment criteria

Neurological score (NS) was performed at different time points after rats were awake, as shown in Table 1. In this study, the 18-point Neurological score (NS) system was used 24 h after modeling, and animals with moderate and severe neurological impairment ranging from 7 to 14 points were selected into the group.

**TABLE 1:** Modified Neurological Severity Score (mNSS) tests and scoring values.

Motor test score values and descriptions	
(Normal score = 0; maximum possible summary score = 6)	
0 or 1^a^	Flexion of forelimb after raising rat by the tail
0 or 1*	Flexion of hindlimb after raising rat by the tail
0 or 1*	Head moved >10° to vertical axis within 30 seconds after raising rat by the tail
0	Normal walk after placing rat on the floor
1	Inability to walk straight after placing rat on the floor
2	Circling toward paretic side after placing rat on the floor
3	Falls down to paretic side after placing rat on the floor
Sensory test score values and descriptions	
(Normal score = 0; maximum possible summary score = 2)	
0 or 1^a^	Placing test (visual and tactile test)
0 or 1^a^	Procioceptive test (deep sensation, pushing paw against table to stimulate limb muscles)
Beam and balance test score values and descriptions	
(Normal score = 0; maximum possible summary score = 6)	
0	Balances with steady posture
1	Grasps side of beam
2	Hugs beam and 1 limb falls down from beam
3	Hugs beam and 2 limbs fall down from beam, or spins on beam (60 seconds)
4	Attempts to balance on beam, but falls off (>40 seconds)
5	Attempts to balance on beam, but falls off (>20 seconds)
6	Falls off; no attempt to balance or hang on to beam (<20 seconds)
Reflex absence and abnormal movements test score values and descriptions	
(Normal score = 0; maximum possible summary score = 4)	
0 or 1^a^	Pinna reflex (head shakes when auditory meatus is touched with cotton)
0 or 1^a^	Corneal reflex (eye blink when cornea is lightly touched with cotton)
0 or 1^a^	Startle reflex (motor response to a brief noise from snapping a clipboard paper)
0 or 1^a^	Seizure, myoclonus, myodystony

^a^ Score value of 1 was given for the inability to perform a test, or for the lack of a tested reflex, or for abnormal movement as described by Chen et al [26].

### The successful establishment of a hemorrhagic stroke model in SD rats was evaluated using MRI

SD rats underwent cranial scanning using a 3.0T S magnetic resonance imaging (MRI) device to assess the hemorrhage status in a model of hemorrhagic stroke. Prior to MRI scanning, the rats were anesthetized via intraperitoneal injection of Sufentanil citrate (Sufentanil) at a dosage of 0.1 mL per 100 g of body weight, ensuring the animals remained sedated and pain-free during the scanning process. The scanning sequence employed was T2-weighted imaging, with a slice thickness of 1 mm. This sequence is particularly sensitive to changes in water content within brain tissue, and acute cerebral hemorrhage areas will appear as hypointense regions. This feature facilitates clear identification of brain edema and hemorrhage areas caused by hemorrhagic stroke, thereby confirming the successful establishment of the cerebral hemorrhage model.

### Amplified culture of AD-MSCs

AD-MSCs were isolated from rats as follows: Approximately 2 g adipose tissue was harvested from the lateral hypogastric region of rat by aseptic surgery. AD-MSCs were isolated following a procedure described previously [27, 28]. Briefly, the adipose tissue was treated with collagenase type I (Life Technologies, Grand Island, NY, United States), and then cultured in α-MEM (Gibco, United States, 41061-029) with 10% FBS (Gibco, United States, 12664025) for 1 day in a T-25 flask (Thermo Fisher, Carlsbad, CA, United States). Floating cells were removed the next day by replacing the medium. Verification of isolated AD-MSCs was performed using antibodies against CD90-BV786, CD105-PE, CD44-PE-CF594, CD29-APC, CD73-BV421, CD14-FITC, CD34-FITC, CD45-FITC, CD31-FITC and HLA-FITC. The isolated AD-MSCs did not express CD14, CD31, CD34, CD45 and HLA. Surface type analysis of the AD-MSCs was performed using a FACSelesta flow cytometer (BD, Germany). The isolated AD-MSCs were propagated for 3 generations within 14 days, and the propagated AD-MSCs were cryopreserved until the use.

### Cell differentiation

The induced differentiation of AD-MSCs was performed by culturing the AD-MSCs in conditioned induction medium (Cyagen, US). The osteogenic differentiation of AD-MSCs was observed by alizarin red staining. Adipogenic differentiation of AD-MSCs was observed by oil Red O staining. The chondrogenic differentiation of AD-MSCs was observed by Alcian blue staining.

### Fluorescent labeling of AD-MSCs by PKH26

AD-MSCs were fluorescently labeled using the PKH26 reagent according to the provided instructions. Reagents were prepared, including 1% human albumin normal saline, complete medium, and PKH26 working liquid (prepared to avoid light and used immediately). AD-MSCs were centrifuged, cleaned, and resuspended in normal saline for sampling and counting. The PKH26 working fluid was prepared according to cell count results, and cells were labeled, stained at room temperature for 5 min with gentle mixing, and then centrifuged. After staining cessation with FBS, AD-MSCs were cleaned, resuspended, passed through a 70 μm cell screen, and centrifuged again. The AD-MSCs concentration was adjusted to 5 × 10^6^ cells/mL using human blood albumin and normal saline, and the cell preparation was stored at 2–8°C for later use. AD-MSCs from 1-month-old and 12-month-old SD rats were prepared similarly for treatment.

### Cell viability assay

LIVE/DEADTM cell imaging kit (Cat No. R37601, Thermo Fisher Scientific, United States) and NucBlueTM live cell stain (Cat No. R37605, Thermo Fisher Scientific, United States) were prepared and added into the medium of AD-MSCs as per manufacturer reference. The cells were then incubated at room temperature for 20 min in darkness. After incubation, the cells were imaged by Nikon A1 laser confocal microscope (Nikon, Japan).

### Caudal venous transfusion of AD-MSCs

The SD rats in the treatment group were treated with AD-MSCs 24 h after intracranial hemorrhage modeling. After the SD rats were fixed with a rat fixation device, the tail vein was disinfected with alcohol, and 5 × 10^6^ cells/mL of AD-MSCs were slowly injected into the rats through the tail vein at a dose of 1 × 10^7^ cells/kg. The injection time is controlled at 1–2 min. During the treatment of ICH rats, AD-MSCs derived from young rats and aged rats were administered at identical doses.

### Neural function score

Neurological function scores were performed 24 h after modeling before treatment, 7, 14, 21 and 28 days after treatment. The neural function scores were mainly from the following four aspects listed in Table 1 [29]: 1. Motor function (6 points): lifting the rat tail and observing the flexion of the contralateral forelimb; The rats were placed on the floor and observed to walk. 2. Sensation test (2 points): mainly deep sense. 3. Balance beam test (6 points): mainly observe the balance of rats after cerebral ischemia, so as to judge the degree of neurological dysfunction. 4. Reflex (4 points): including auricle, cornea, fright, convulsive reflex, etc. 0 is normal, 18 is the most serious. A score of 1-6 indicates mild damage; 7-12 points indicate moderate damage; A score of 13–18 indicates serious damage.

### Euthanasia of rats using carbon dioxide

Rats were euthanized using carbon dioxide. The rats are placed in a transparent, airtight anesthetic chamber, and the valve of the carbon dioxide tank is slowly opened to fill the chamber with carbon dioxide at a flow rate of 10%–30% of the container’s volume per minute for 3–5 min. The rats will initially exhibit agitation, followed by gradual anesthesia, collapse, and cessation of breathing. After stopping the gas supply, the rats are observed for an additional 2–3 min to confirm death by palpating the chest for a heartbeat and observing for the absence of respiration. Immediately after confirming the animals' death, they are dissected for tissue collection.

### Immunohistochemistry/immunofluorescence staining

HE and immunohistochemical tests were performed 28 days after treatment to evaluate the therapeutic effect of allogeneic young and old AD-MSCs. The collected rat brain tissue was fixed with 4% paraformaldehyde, embedded in paraffin by the usual method, sliced to a thickness of 4 μm, stained with HE and Masson according to the manufacturer’s instructions. All sections were histologically analyzed and photographed with an optical microscope.

The homing of AD-MSCs at brain injury sites was observed by immunofluorescence (PKH26) detection at 24 h, 48 h and 72 h after AD-MSCs treatment.

### Rat derived AD-MSCs and brain tissue single cell RNA-seq library generation and sequencing

The brain tissue of the infarct margin area was collected from rat with a scalpel (or surgical scissors). Under sterile conditions, wash twice with pre-cooled RPMI 1640 + 0.04% BSA medium. The tissue was fully cut into small pieces of about 0.5 mm^3^ with surgical scissors and put into freshly prepared enzymatic solution (enzymatic solution composition, concentration, brand), and digested by enzymatic digestion in a constant temperature incubator at 37°C for 30–60 min (references), and mixed inversely every 5–10 min. The digested cell suspension was filtered by BD 40 μm cell screen for 1-2 times, centrifuged at 4°C and 300 g for 5 min. After the precipitation was suspended with appropriate medium, the same volume of red blood cell lysate (MACS, No. 130-094–183) was added, mixed, left for 10 min at 4°C, cell suspension 300 g centrifuged for 5 min, and the supernatant was discarded. The precipitation was washed in the medium once, centrifuged at 300 g for 5 min, and the supernatant was discarded. The cell precipitation was suspended in the medium of 100 μL, and the cell concentration and viability were calculated by Luna cell counter.

The freshly prepared single-cell suspension was adjusted to 700-1,200 cell/μL according to the 10 × Genomics Chromium Next GEM Single Cell 3ʹ Reagent Kits v3.1 (No. 1000268) Operation manual for computer and library construction. The constructed library was sequenced using Illumina Nova 6000 PE150 platform.

### Single cell RNA-seq data pre-processing and quality control

Single-cell RNA sequencing data generated using the 10x Genomics platform was processed with Cell Ranger version 8.0.0 (10x Genomics). Raw sequencing reads were aligned to the rat reference genome and assigned to individual cells of origin according to the cell-specific barcodes using the Cell Ranger pipeline (10× Genomics). The rat genome used in this analysis was obtained from the NCBI database, specifically version GCF_015227675.2. The output gene expression matrix was then imported into the Seurat (v4.3.0) R package for downstream analysis. SoupX R package (v1.6.2) was employed to removes ambient RNA contamination from droplet-based single-cell RNA sequencing data. DoubletFinder R package (v2.0.4) was employed to identify the poorly dissociated doublets in the single-cell suspension preparation process. Low-quality cells (<200 genes/cell and >10% mitochondrial genes) were excluded. Low-quality genes detected in less three cells were excluded. Gene expression levels for each cell were normalized by total expression, multiplied by a scale factor (10,000), and log-transformed. Top 2000 highly variable features (HVGs) in a sample were identified by “FindVariableFeatures” Seurat function. Brain samples and AD-MSCs samples were merged respectively, and variable features per sample were merged correspondingly. Gene expression data was scaled according to UMI number using the “ScaleData” Seurat function, meanwhile, nUMI and percent.mito were regressed out, ensuring that each gene’s expression is centered and scaled to facilitate downstream dimensionality reduction and clustering analyses. We reduced the dimensionality of the data by performing the principal component analysis (PCA) on HVGs. Harmony R package (v0.1.1) was used to remove batch effect. To display data, the Unsupervised Uniform Manifold Approximation and Projection (UMAP) was applied to cell loadings of selected PCs. The “FindClusters” Seurat function was employed to identify distinct cell clusters based on gene expression patterns, using a shared nearest neighbor (SNN) modularity optimization algorithm to group cells with similar profiles. To identify differentially expressed genes between two clusters, we used the “find.markers” Seurat function with logfc. threshold = 0.25 and test. use = “wilcox”.

Subcluster identification steps was same to the above cluster identification steps.

### Celltype indentification

For brain samples, we divided cells into 31 clusters using the “FindClusters” Seurat function in with resolution 0.6. We identified 18 cell types including two unknown populations. The identified cell types are: Astrocytes (Gfap, Aqp4, Rorb), Microglial cells (Tmem119, P2ry12, Cx3cr, Sall), Oligodendrocytes (Mbp, Plp1, Mog, Olig1, Olig2), Neurons (Gria1, Syt1), OPCs (Vcan, Pdgfra, Sox10), Neuroblasts (Ccnd2, Dcx, Sox4, Sox11, Stmn2), T cells (Cd3e, Cd3g), Monocytes (Cd74, Plac8, Ccr2), Macrophages (Cd14, Cd68, Lyz2, Gpnmb), Myeloid derived cells (Lilrb4), Neutrophils (S100a9, S100a8), Endothelial cells (Vwf, Pecam1), SMCs (Myh11, Acta2), Pericytes (Pdgfrb, Vtn, Cspg4), Fibroblasts (Col1a1, Col1a2, Col3a1), Epithelial cells (Kl, Folr1, Sostdc1), Ependymal cells (Ccdc153, Tmem212, Dnah11), Cycling cells (Top2a, Mki67).

### Enrichment analysis

Enriched KEGG terms of marker genes were identified using the enrichKEGG function from the clusterProfiler package (v4.6.0), with gene annotations provided by the org.Rn.eg.db database in the org.Rn.eg.db R package (v3.16.0).

### Statistical analysis

Data were obtained from three separate experiments and expressed as means ± standard error of the mean (SEM). A single factor design was applied to this study. After a significant interaction was detected by the analysis of variance (SPSS), the significance of the main effects was further determined by T-test (2 groups) and Kruskal-Wallis test (3 groups). The Mann-Whitney U test (Bonferroni correction) was used for *post hoc* statistical testing. The level of significance was considered when *P* < 0.05.

## Results

### AD-MSCs significantly promote the repair of damaged brain tissues in ICH rats

**FIGURE 1:**
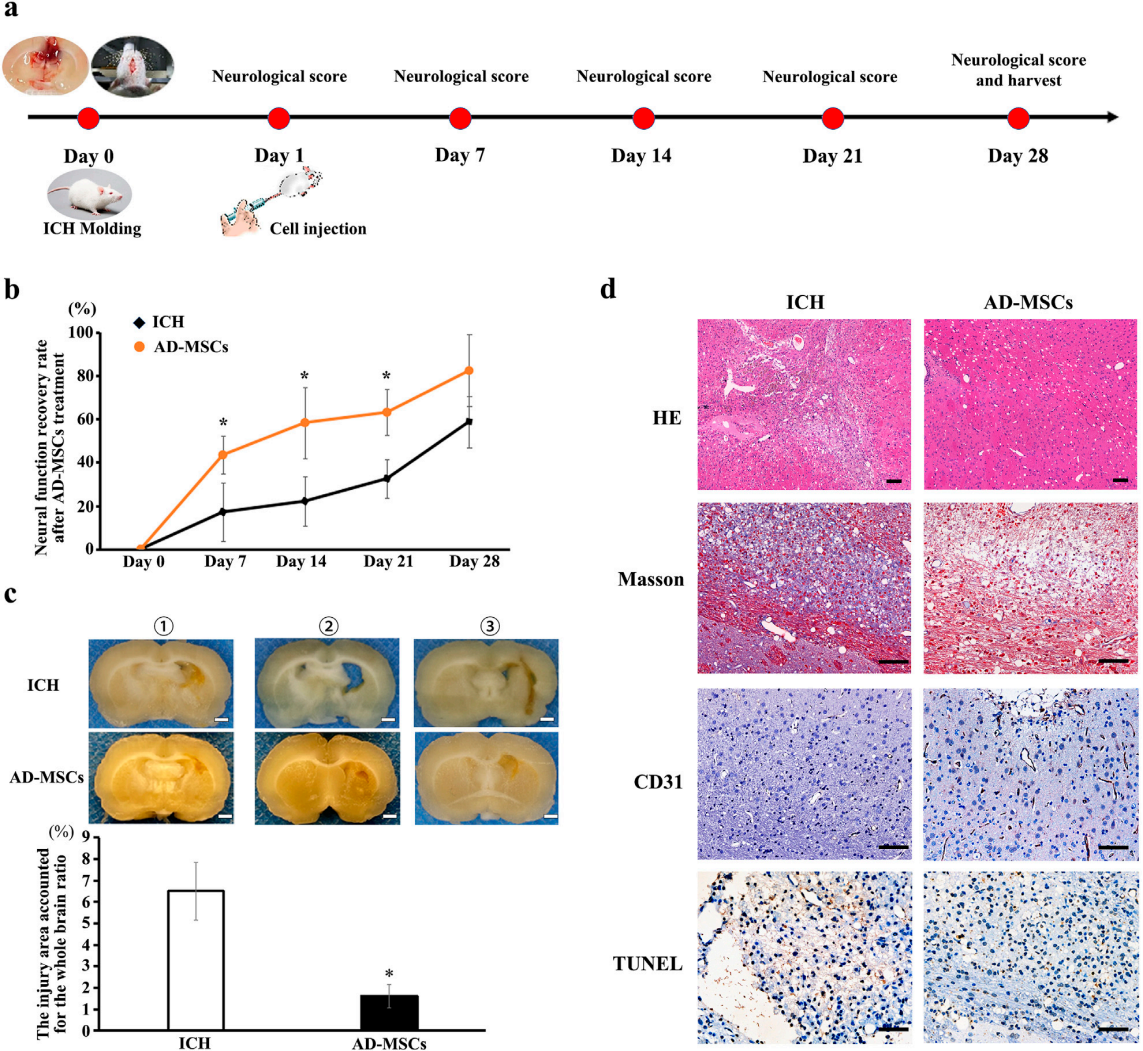
Intravenous infusion of allogeneic AD-MSCs significantly inhibited brain injury 24 h after ICH modeling. **(a)** Experimental procedure of treating ICH rats with AD-MSCs. **(b)** Neural function scores of rats at different time points after AD-MSCs treatment (ICH group: n = 6, AD-MSCs group: n = 5). **(c)** General observation of brain tissue. * indicated significant difference compared with the control group (P < 0.05). Bar = 1 mm. ①, ②, ③ represent different experimental batches. **(d)** HE staining and immunohistochemical staining of brain tissue were performed. HE results indicated the gross structural changes after brain tissue injury and AD-MSCs treatment. Masson test results indicated the degree of fibrosis in brain tissue. CD31 test results indicate the formation of blood vessels in brain tissue. TUNEL test results indicate the apoptosis of neurons in brain tissue. Bar = 200 μm.

The rat model of ICH was established by intraventricular injection of collagenase (Figure 1A), the result of nuclear magnetic test 24 h after operation showed that the mold was made successfully (Supplementary Figure S1). The neural function score indicated a significant loss of neural function in the rats following ICH modeling, which gradually recovered to a certain extent during the subsequent 28-day observation period (ICH group: n = 6). Compared to the ICH group, rats treated with AD-MSCs exhibited more pronounced recovery of neural function (Figure 1B). (AD-MSCs group: n = 5). The gross observation results of brain tissue sampling showed that there were significant defects in the rat brain tissue after ICH modeling, and AD-MSCs treatment significantly limited the expansion of the damage (Figure 1C). Further histopathological results showed that, compared with the ICH group, AD-MSCs treatment significantly reduced collagen formation and apoptosis in the injured brain tissue, and significantly increased the formation of blood vessels (Figure 1D). These results showed that the treatment of AD-MSCs significantly promote the repair of damaged brain tissue and recover the nerve function. Next, we analyzed the effects of AD-MSCs by single-cell sequencing.

### AD-MSCs promote the damaged brain tissues repair by increasing and activating the resident microglia

**FIGURE 2:**
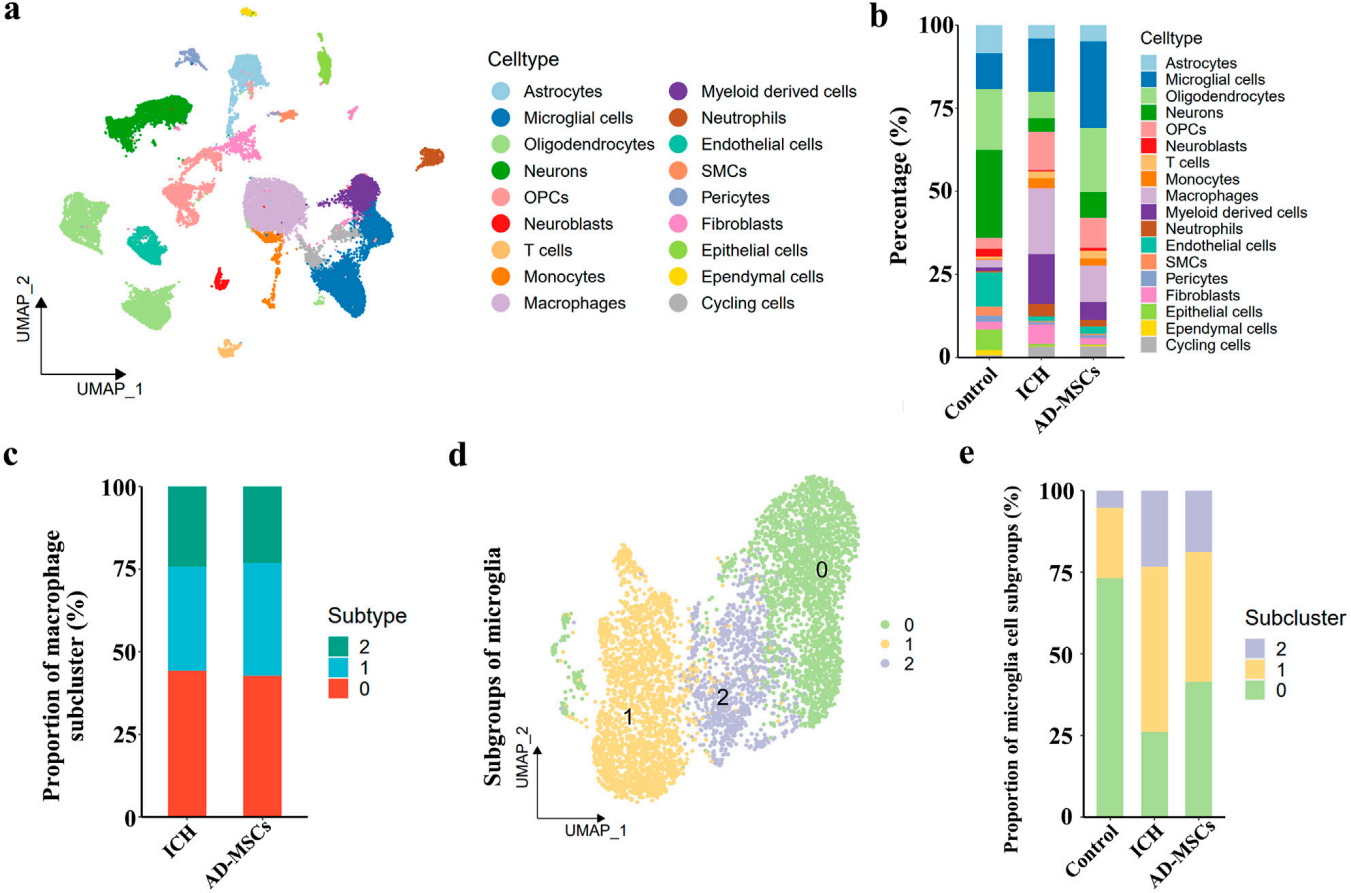
Single cell transcriptome sequencing and analysis of brain tissue. **(a)** The analysis of brain tissue cells based on unsupervised clustering showed that brain tissue cells could be divided into 18 categories. **(b)** The classification of each cell subgroup was identified by characteristic markers. **(c)** Subpopulation ratio analysis of macrophages in ICH group and AD-MSCs treatment group. **(d)** Subpopulation analysis of microglial cells in brain tissue. **(e)** The proportion of microglia subsets in the normal control group, ICH group and AD-MSCs treatment group.

After 3 days of AD-MSCs treatment, brain tissues were sampled and sequenced by 10×Genomics. The brain tissue cells were divided into 18 subgroups by unsupervised clustering. The classification of each cell subgroup was identified by characteristic markers (Supplementary Figure S2), and the results were shown in Figure 2A. Compared with the ICH group, AD-MSCs treatment significantly reduced the proportion of fibroblasts and moderately increased the proportion of endothelial cells as well as the proportion of neuronal cells (Figure 2B).

**FIGURE 3:**
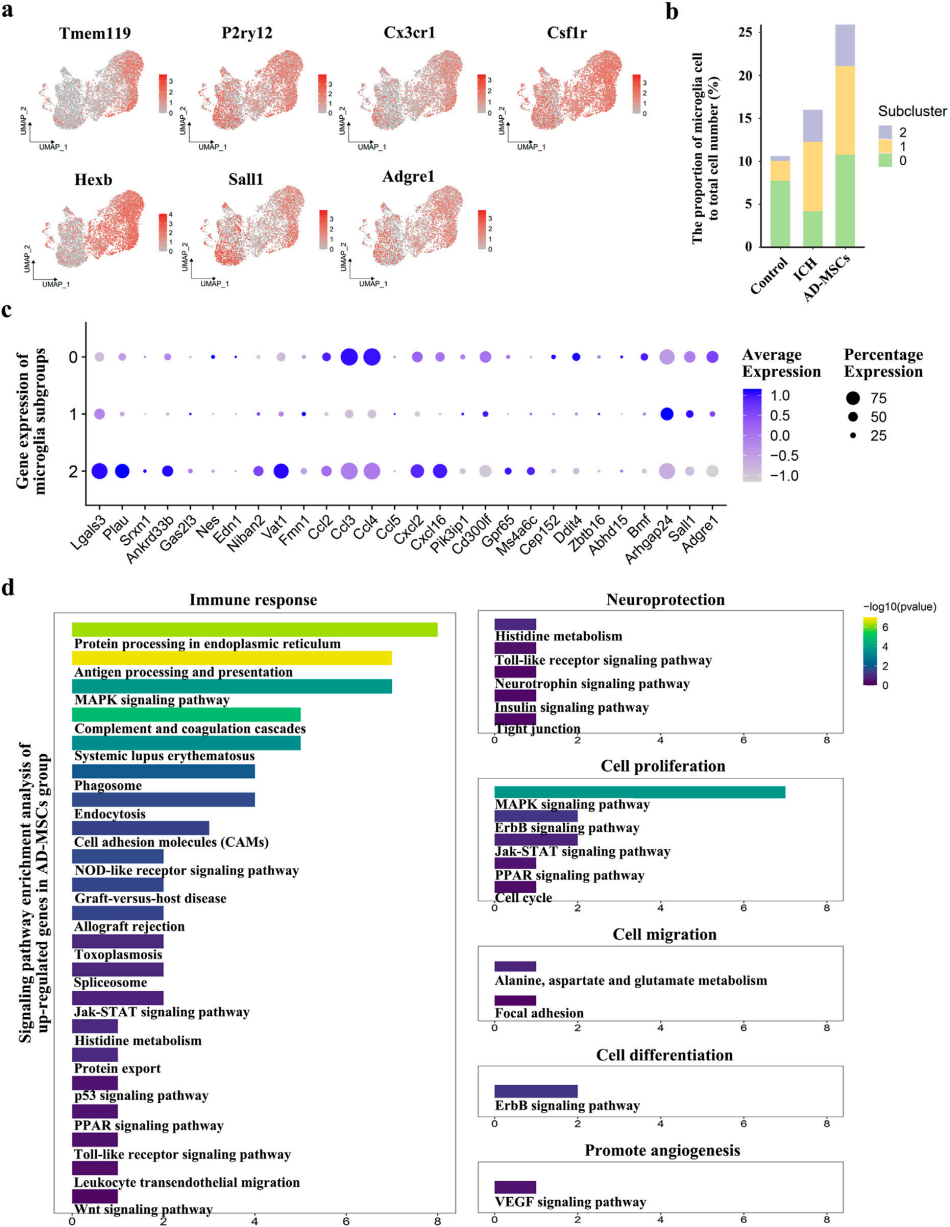
Based on the results of single cell transcriptome sequencing, the categories and functions of microglia subsets were analyzed. **(a)** The expression of genes related to resting state of microglia and genes specific to resident microglia in microglia subgroups. **(b)** The proportion of microglia and their subsets in the total cell volume of brain tissue in the normal control group, ICH group and AD-MSCs treatment group. **(c)** Expression of genes associated with inflammation and anti-inflammatory in various subpopulations of microglia. **(d)** Signaling pathway enrichment analysis of genes that cause microglial hyperexpression after AD-MSCs treatment compared with ICH group.

In terms of the effect on immune cells, AD-MSCs significantly reduced the invasion of mononuclear/macrophage cells. However, compared with the ICH group, AD-MSCs did not affect the transformation of macrophage subgroups (Figure 2C). In addition, AD-MSCs significantly increased the proportion of microglia (Figure 2B). It is generally believed that the increase of microglia leads to increased brain tissue damage. Therefore, we conducted an in-depth analysis of the increase of microglia induced by AD-MSCs. Unsupervised cluster analysis showed that microglia could be divided into three subgroups (Figure 2D). In order to explore the function of these three subgroups, we first detected the expression of microglia cells homeostasis genes Tmem119, P2ry12, Csf1r, Cx3cr1 and Hexb in each subpopulation. The results showed that these genes were highly expressed in subpopulations 0 (Figure 3A), which constituted 70% of the total microglia population in the control group (Figure 2E). Therefore, we determined that subgroup 0 was a resting microglia. We further analyzed subpopulations 1 and 2 of microglia. It has been proved that there are active microglia with anti-inflammatory and pro-inflammatory phenotypes in injured brain tissue [30]. Therefore, we examined the expression of related genes in microglia cells subgroups 1 and 2. The results showed that the genes closely related to proinflammation were highly expressed in subpopulation 2, including Lgals3, Plau, Ankrd33b, Fam129b, Vat1, Ccl2, Ccl3, Ccl4, Cxcl2 and Cxcl16, however, which were not expressed in subpopulation 1 (Figure 3C). It suggested that subpopulation 1 and subpopulation 2 are two completely different activated microglia cells.

Studies have shown that when brain tissue injured, the recruited monocytes can also transform into microglia, which mainly play a pro-inflammatory role [31]. Therefore, we detected the expression of the Sall1 and Adgre1, which are characteristic microglia-resident gene, in subgroup 1 and subgroup 2. The results showed that Sall1 and Adgre1, which were specifically expressed only in resident microglia, were only highly expressed in subgroup 1 (Figure 3A). Thus, the proinflammatory phenotype of subgroup 2 may be derived from the transformation of bone marrow-derived monocyte, while the anti-inflammatory subgroup 1 is derived from resident microglia. We further examined the proportion of 3 microglia subgroups in each rat group. The results showed that brain injury significantly increased the proportion of activated microglia, including the conversion of recruited monocytes into microglia. The treatment with AD-MSCs significantly increased the overall ratio of microglia in brain tissue. Moreover, the increase in activated microglia was primarily derived from resident microglia and displayed an anti-inflammatory phenotype (Figure 3B). The signaling pathway enrichment of the up-regulated genes in microglia was further analyzed. The results demonstrated that AD-MSCs treatment significantly enhanced the expression of microglial signaling pathways associated with cellular proliferation, immune response, and neuroprotection compared to the ICH group (Figure 3D). Therefore, changes in the proportion and subgroups of microglia may be the root cause of AD-MSCs limiting brain tissue damage.

### Csf1^+^ AD-MSCs are the key to the regulation of microglia

**FIGURE 4:**
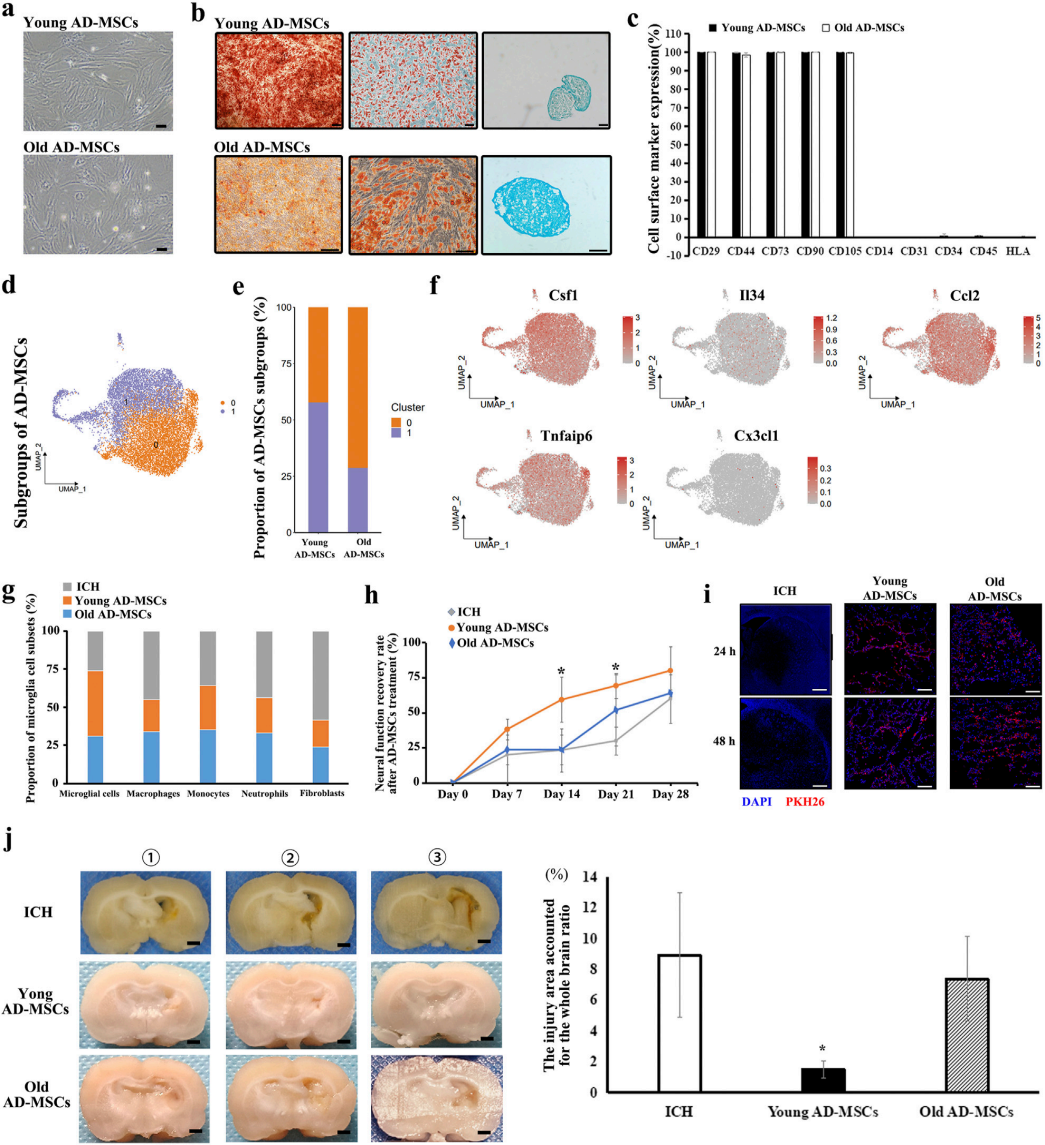
Therapeutic effect of Young AD-MSCs and Old AD-MSCs on ICH rats and analysis of key cell subgroups. **(a)** Cell morphology of Young AD-MSCs and Old AD-MSCs expanded *in vitro*. Bar = 200 μm. **(b)** Induction differentiation of Young AD-MSCs and Old AD-MSCs into osteoblasts, lipoblasts and chondroblasts. Bar = 100 μm. **(c)** Surface marker detection of Young AD-MSCs and Old AD-MSCs by flow cytometry. **(d, e)** Subpopulation analysis of Young AD-MSCs and Old AD-MSCs was performed based on single-cell transcriptome sequencing. **(f)** Expression of genes closely related to microglia proliferation, chemotaxis and phenotypic transformation in AD-MSCs subgroups. **(g)** Effects of Young AD-MSCs and Old AD-MSCs on the proportion of immune-associated cells and fibroblasts in brain tissue. **(h)** Neural function scores of Young AD-MSCs and Old AD-MSCs treated rats (n = 4 for each group). **(i)** Homing detection of PKH26-labeled Young AD-MSCs and Old AD-MSCs in damaged brain tissue of rats. **(j)** General observation of brain tissue (n = 4 for each group). Bar = 1 mm * indicated significant difference compared with the control group (P < 0.05). (n = 3 for each group) ①, ②, ③ represent different experimental batches.

We administered various qualities of AD-MSCs to ICH rats in order to investigate the mechanism of action of AD-MSCs in promoting damaged brain tissue repair through microglia regulation. AD-MSCs obtained from 1-month-old rats (termed young AD-MSCs) and 12-month-old rats (termed old AD-MSCs) were expanded and cultured *in vitro*, followed by single-cell transcriptome sequencing analysis. The results showed that despite the identical viability, surface markers and capacity for differentiation into osteoblasts, lipoblasts, and chondroblasts between young AD-MSCs and old-AD-MSCs (Figures 4A–C; Supplementary Figure S3), significant disparities were observed at the transcriptome level. AD-MSCs were divided into two subgroups, and the proportion of subgroup 1 of young AD-MSCs was significantly higher than that of old AD-MSCs (Figures 4D,E). We examined the expression of genes related to microglia proliferation, homeostasis maintenance and chemotaxis in different subgroups of AD-MSCs. The results showed that there was no significant difference in the expression of Il34, Ccl2, Tnfaip6 and Cx3cl1 between the two AD-MSCs subgroups, while Csf1 was highly expressed in subgroup 1 of AD-MSCs (Figure 4F), which suggests that the expression of Csf1 in young AD-MSCs is significantly higher than that in Old AD-MSCs. Since Csf1 plays an important role in promoting the proliferation of microglia, we treated ICH rats with these two types of cells respectively to observe their effects on microglia and the therapeutic effect on injured brain tissue.

Young AD-MSCs and old AD-MSCs were injected through the caudal vein 24 h after ICH modeling. Single cell sequencing of rat brain tissue showed that young AD-MSCs significantly increased the number of microglia and inhibited the number of monocyte-derived macrophages and fibroblasts compared with ICH group. The promotion effect of old AD-MSCs on microglia cells and the inhibition effect on monocyte-derived macrophages were significantly weaker than that of young AD-MSCs (Figure 4G). We further evaluated the therapeutic effects of two types of AD-MSC. Neural function scores showed that young AD-MSCs could significantly restore the neural function of ICH rats, while old AD-MSCs had no therapeutic effect (Figure 4H). The histopathological results showed that young AD-MSCs significantly inhibited the expansion of injury, while the brain injury of old AD-MSCs was consistent with that of the ICH group, and had no therapeutic effect (Figure 4J). Homing of AD-MSCs is an important factor in determining the therapeutic effect of AD-Mscs. We used PKH26-labeled AD-MSCs to perform reinfusion at 24 h after ICH modeling, and the homing of cells in brain tissue was simplified at 24 h and 48 h after reinfusion. The results showed that there was no significant difference in homing ability between young AD-MSCs and old AD-MSCs (Figure 4I).

## Discussion

In this study, we utilized an ICH rat model to investigate the mechanism of action of AD-MSCs in the treatment of stroke. Our results demonstrate that AD-MSCs exert their therapeutic effects by regulating the number and phenotype of resident microglia, thereby promote the repair of damaged brain tissues.

As resident macrophage cells, microglia are the principal immune cells of the brain, and the first to respond to the pathophysiological changes induced by stroke. Previous research has suggested that microglia activation leads to exacerbated neural tissue damage. In this study, however, we observed that AD-MSCs facilitate the proliferation and activation of microglia, thereby mitigating brain tissue damage. Following brain tissue injury, monocytes are recruited to the injured site and contribute to the inflammatory response. It has been demonstrated that monocytes can differentiate into microglia-like cells, predominantly expressing pro-inflammatory cytokines that exacerbate neural impairment. In this study, microglia in injured brain tissue transition from a quiescent to an activated state, and nearly 50% of activated microglia are derived from monocytes. AD-MSCs significantly inhibit the infiltration of monocytes. More importantly, AD-MSCs treatment notably increases the proportion of innately developed resident microglia while inhibiting the transformation of recruited monocytes into microglia. This increase in the proportion of resident microglia, along with an increase in anti-inflammatory phenotypes, is closely associated with the therapeutic effect of AD-MSCs in inhibiting brain tissue damage. When AD-MSCs derived from 12-month-old rats were used for treatment, they were ineffective in increasing microglia numbers and inhibiting the number of recruited monocytes/macrophages, resulting in poor therapeutic outcomes. Therefore, increasing the number of innately developed resident microglia in brain tissue after injury may be an important direction, the underlying molecular mechanisms need further elaboration. Additionally, the correlation between AD-MSCs increasing the number of resident microglia and inhibiting the infiltration of monocyte-derived macrophages remains unclear, and the relevant molecular mechanisms should be investigated in future work.

The heterogeneity of *in vitro* cultured MSCs may significantly contribute to the inconsistent clinical therapeutic outcomes observed in stroke treatment [32, 33]. This heterogeneity refers to the variations in morphology, protein expression, transcriptome expression, staining characteristics, ultrastructure, and function among individual cells within primary MSC cultures. These variations are influenced by factors such as donor age and health status, tissue type, tissue microenvironment, and *in vitro* culturing techniques [34]. As a result, primary MSC cultures contain multiple cell subgroups with distinct functions. Studies have demonstrated that these different cell subgroups exhibit unique biological functions [35, 36]. Therefore, identifying functional subgroups of MSCs is crucial for the development of effective stroke treatments. In this study, single-cell transcriptome sequencing revealed distinct cell subpopulations in AD-MSCs derived from 1-month-old and 12-month-old rats. Compared to AD-MSCs from older rats, those from younger rats had a higher proportion of the Csf1 subpopulation, which is closely associated with microglial cell proliferation. *In vivo* studies showed that AD-MSCs from 1-month-old rats significantly promoted an increase in resident microglia cell within injured brain tissue and facilitated nerve function recovery. Consequently, enhancing the proportion of AD-MSCs expressing high levels of Csf1 may be pivotal in ensuring the therapeutic efficacy of stroke treatment products during their development.

This study has several limitations. Specifically, we only investigated the effects of AD-MSC transfusions administered 24 h after the induction of ICH modeling. It remains unclear whether treatments administered at different time points following modeling (e.g., 3, 7, or 21 days post-injury) would exhibit comparable therapeutic efficacy and operate through similar mechanisms. Furthermore, the precise mechanisms underlying the homing of MSCs to injured tissue and their subsequent promotion of resident microglia proliferation following injury warrant further investigation.

## Conclusion

This study investigated the interaction between AD-MSCs and microglia in ICH models, as well as their therapeutic impact on damaged brain tissue. Our findings revealed that intravenously injected allogeneic AD-MSCs inhibited the infiltration of recruited monocytes by stimulating an increase in resident microglia and promoting an anti-inflammatory phenotype transformation within injured brain tissue, thereby promote the repair of damaged tissues. A subgroup of AD-MSCs expressing Csf1 emerged as a crucial functional component in stroke treatment. Future research will focus on elucidating the mechanisms underlying the enhancement of resident microglia by AD-MSCs and its subsequent influence on the damage repair process.

## Data Availability

The datasets presented in this study can be found in online repositories. The names of the repository/repositories and accession number(s) can be found in the article/Supplementary Material.

## References

[1] Feigin VL Lawes CMM Bennett DA Barker-Collo SL Parag V. Worldwide stroke incidence and early case fatality reported in 56 population-based studies: a systematic review. The Lancet Neurol (2009) 8:355–69. 10.1016/s1474-4422(09)70025-0 19233729

[2] An SJ Kim TJ Yoon BW. Epidemiology, risk factors, and clinical features of intracerebral hemorrhage: an update. J Stroke (2017) 19(1):3–10. 10.5853/jos.2016.00864 28178408 PMC5307940

[3] Quertainmont R Cantinieaux D Botman O Sid S Schoenen J Franzen R. Mesenchymal stem cell graft improves recovery after spinal cord injury in adult rats through neurotrophic and pro-angiogenic actions. PLoS ONE (2012) 7(6):e39500. 10.1371/journal.pone.0039500 22745769 PMC3380009

[4] Tetzlaff W Okon EB Karimi-Abdolrezaee S Hill CE Sparling JS Plemel JR A systematic review of cellular transplantation therapies for spinal cord injury. J neurotrauma (2011) 28(SP8):1611–82. 10.1089/neu.2009.1177 20146557 PMC3143488

[5] Chung JW Chang WH Bang OY Moon GJ Kim SJ Kim SK Efficacy and safety of intravenous mesenchymal stem cells for ischemic stroke. Neurology (2021) 96(7):e1012–e1023. 10.1212/wnl.0000000000011440 33472925

[6] Law ZK Tan HJ Chin SP Wong CY Wan Yahya WNN Muda AS The effects of intravenous infusion of autologous mesenchymal stromal cells in patients with subacute middle cerebral artery infarct: a phase 2 randomized controlled trial on safety, tolerability and efficacy. Cytotherapy (2021) 23(9):833–40. 10.1016/j.jcyt.2021.03.005 33992536

[7] de Celis-Ruiz E Fuentes B Alonso de Leciñana M Gutiérrez-Fernández M Borobia AM Gutiérrez-Zúñiga R Final results of allogeneic adipose tissue–derived mesenchymal stem cells in acute ischemic stroke (AMASCIS): a phase II, randomized, double-Blind, Placebo-Controlled, Single-Center, pilot clinical trial. Cell Transplant (2022) 31:09636897221083863. 10.1177/09636897221083863 35301883 PMC8943307

[8] Bernardo M Fibbe W. Mesenchymal stromal cells: sensors and switchers of inflammation. Cell stem cell (2013) 13(4):392–402. 10.1016/j.stem.2013.09.006 24094322

[9] Lu D Xu Y Liu Q Zhang Q. Mesenchymal stem cell-macrophage crosstalk and maintenance of inflammatory microenvironment homeostasis. Front Cel Dev Biol (2021) 9:681171. 10.3389/fcell.2021.681171 PMC8267370 34249933

[10] Colonna M Butovsky O. Microglia function in the central nervous System during health and neurodegeneration. Annu Rev Immunol (2017) 35(35):441–68. 10.1146/annurev-immunol-051116-052358 28226226 PMC8167938

[11] Alsbrook DL Di Napoli M Bhatia K Biller J Andalib S Hinduja A Neuroinflammation in acute ischemic and hemorrhagic stroke. Curr Neurol Neurosci Rep (2023) 23(8):407–31. 10.1007/s11910-023-01282-2 37395873 PMC10544736

[12] Borst K Dumas AA Prinz M. Microglia: immune and non-immune functions. Immunity (2021) 54(10):2194–208. 10.1016/j.immuni.2021.09.014 34644556

[13] Li Y Dong Y Ran Y Zhang Y Wu B Xie J Three-dimensional cultured mesenchymal stem cells enhance repair of ischemic stroke through inhibition of microglia. Stem Cell Res Ther (2021) 12(1):358. 10.1186/s13287-021-02416-4 34154653 PMC8218508

[14] Liu Y Zeng R Wang Y Huang W Hu B Zhu G Mesenchymal stem cells enhance microglia M2 polarization and attenuate neuroinflammation through TSG-6. Brain Res (2019) 1724. 10.1016/j.brainres.2019.146422 31472111

[15] Noh MY Lim SM Oh KW Cho KA Park J Kim KS Mesenchymal stem cells modulate the functional properties of Microglia *via* TGF-β secretion. Stem Cells Translational Med (2016) 5(11):1538–49. 10.5966/sctm.2015-0217 PMC5070497 27400795

[16] Zanier ER Pischiutta F Riganti L Marchesi F Turola E Fumagalli S Bone marrow mesenchymal stromal cells drive protective M2 microglia polarization after brain trauma. Neurotherapeutics (2014) 11(3):679–95. 10.1007/s13311-014-0277-y 24965140 PMC4121458

[17] Liang ZH Gu JJ Yu WX Guan YQ Khater M Li XB. Bone marrow mesenchymal stem cell transplantation downregulates plasma level and the microglia expression of transforming growth factor β1 in the acute phase of cerebral cortex ischemia. Chronic Dis Translational Med (2020) 6:270–80. 10.1016/j.cdtm.2020.05.005 PMC7729118 33336172

[18] Li X Huang M Zhao R Zhao C Liu Y Zou H Intravenously delivered allogeneic mesenchymal stem cells bidirectionally regulate inflammation and induce neurotrophic effects in distal middle cerebral artery occlusion rats within the first 7 days after stroke. Cell Physiol Biochem (2018) 46:1951–70. 10.1159/000489384 29719282

[19] Xu C Fu F Li X Zhang S. Mesenchymal stem cells maintain the microenvironment of central nervous system by regulating the polarization of macrophages/microglia after traumatic brain injury. Int J Neurosci (2017) 127(12):1124–35. 10.1080/00207454.2017.1325884 28464695

[20] Liu W Yu M Xie D Wang L Ye C Zhu Q Melatonin-stimulated MSC-derived exosomes improve diabetic wound healing through regulating macrophage M1 and M2 polarization by targeting the PTEN/AKT Pathway. Stem Cell Res and Ther (2020) 11:259. 10.1186/s13287-020-01756-x 32600435 PMC7322868

[21] Fernández-López D Faustino J Klibanov AL Derugin N Blanchard E Simon F Microglial cells prevent hemorrhage in neonatal focal arterial stroke. The J Neurosci (2016) 36(10):2881–93. 10.1523/jneurosci.0140-15.2016 26961944 PMC4783493

[22] Ransohoff RM. A polarizing question: do M1 and M2 microglia exist? Nat Neurosci (2016) 19:987–91. 10.1038/nn.4338 27459405

[23] Masuda T Sankowski R Staszewski O Prinz M. Microglia heterogeneity in the single-cell era. Cell Rep (2020) 30:1271–81. 10.1016/j.celrep.2020.01.010 32023447

[24] Haast RA Gustafson DR Kiliaan AJ. Sex differences in stroke. J Cereb Blood Flow Metab (2012) 32(12):2100–7. 10.1038/jcbfm.2012.141 23032484 PMC3519418

[25] Tang T Hu L Liu Y Fu X Li J Yan F Sex-associated differences in neurovascular dysfunction during ischemic stroke. Front Mol Neurosci (2022) 15:860959. 10.3389/fnmol.2022.860959 35431804 PMC9012443

[26] Li Y Zhang J. Animal models of stroke. Anim Models Exp Med (2021) 4:204–19. 10.1002/ame2.12179 PMC8446711 34557647

[27] Lee RH Kim B Choi I Kim H Choi HS Suh K Characterization and expression analysis of mesenchymal stem cells from human bone marrow and adipose tissue. Cell Physiol Biochem (2004) 14(4-6):311–24. 10.1159/000080341 15319535

[28] Zuk PA Zhu M Mizuno H Huang J Futrell JW Katz AJ Multilineage cells from human adipose tissue: implications for cell-based therapies. Tissue Eng (2001) 7(2):211–28. 10.1089/107632701300062859 11304456

[29] Chen J Sanberg PR Li Y Wang L Lu M Willing AE Intravenous administration of human umbilical cord blood reduces behavioral deficits after stroke in rats. Stroke (2001) 32(11):2682–8. 10.1161/hs1101.098367 11692034

[30] Li H Liu P Zhang B Yuan Z Guo M Zou X Acute ischemia induces spatially and transcriptionally distinct microglial subclusters. Genome Med (2023) 15:109. 10.1186/s13073-023-01257-5 38082331 PMC10712107

[31] Chen HR Chen CW Kuo YM Chen B Kuan IS Huang H Monocytes promote acute neuroinflammation and become pathological microglia in neonatal hypoxic-ischemic brain injury. Theranostics (2022) 12(Issue 2):512–29. 10.7150/thno.64033 34976198 PMC8692901

[32] Mendicino M Bailey A Wonnacott K Puri R Bauer S. MSC-Based product characterization for clinical trials: an FDA perspective. Cell Stem Cell (2014) 14(2):141–5. 10.1016/j.stem.2014.01.013 24506881

[33] Dent E Martin FC Bergman H Woo J Romero-Ortuno R Walston JD. Management of frailty: opportunities, challenges, and future directions. The Lancet (2019) 394(10206):1376–86. 10.1016/s0140-6736(19)31785-4 31609229

[34] Costa LA Eiro N Fraile M Gonzalez LO Saá J Garcia-Portabella P Functional heterogeneity of mesenchymal stem cells from natural niches to culture conditions: implications for further clinical uses. Cell Mol Life Sci (2021) 78(2):447–67. 10.1007/s00018-020-03600-0 32699947 PMC7375036

[35] Li X Guo W Zha K Jing X Wang M Zhang Y Enrichment of CD146+ adipose-derived stem cells in combination with articular cartilage extracellular matrix scaffold promotes cartilage regeneration. Theranostics (2019) 9(17):5105–21. 10.7150/thno.33904 31410204 PMC6691381

[36] Liang ZX Liu HS Xiong L Zeng ZW Zheng XB Kang L GAS6 from CD200+ adipose-derived stem cells mitigates colonic inflammation in a macrophage-dependent manner. J Crohn's Colitis (2023) 17(2):289–301. 10.1093/ecco-jcc/jjac123 36006655

