# Supplementary material for: Csf1+ AD-MSCs promote stroke repair by activating the resident microglia
Source: Exp Biol Med (Maywood). 2025 Aug 20;250:10611. doi: 10.3389/ebm.2025.10611 (PMC12405005; doi:10.3389/ebm.2025.10611)
Supplement: Supplementary file 1 [file DataSheet1.docx]

**Csf1^+^ AD-MSCs promote stroke repair by activating the resident microglia**

Jiguang Hou^1#^, Sunfu Zhang^2#^ Shuang Luo^3^, Xiao Zuo^4^, Fei Ma^4^, Huizhen Wang^4^, Pengfei Han^4^, Ping Zhu^4^, Ning Wang^4^ Xiaoming Hou^5^, Jin Li^1^*

^1^ Department of Neurosurgery, West China Hospital of Sichuan University, Chengdu, China.

^2^ Department of Neurosurgery, Third People's Hospital of Chengdu, China.

^3^ Department of Neurosurgery, Fifth People's Hospital of Chengdu, China.

^4^ Tasly Stem Cell Biology Laboratory, Tasly Group, Tianjin, China.

^5^ Regenerative Medicine Research Center, West China Hospital of Sichuan University, Chengdu, China.

#Jiguang Hou and Sunfu Zhang made equal contributions to this work

Running title: AD-MSCs therapy for stroke

*Correspondence to: Jin Li

Department of Neurosurgery, West China Hospital of Sichuan University, Chengdu, China. No. 37 Guoxue Alley, Wuhou District, Chengdu 610041, China.

Email address: jimlijin76@126.com

**Supplment Figure 1**

**
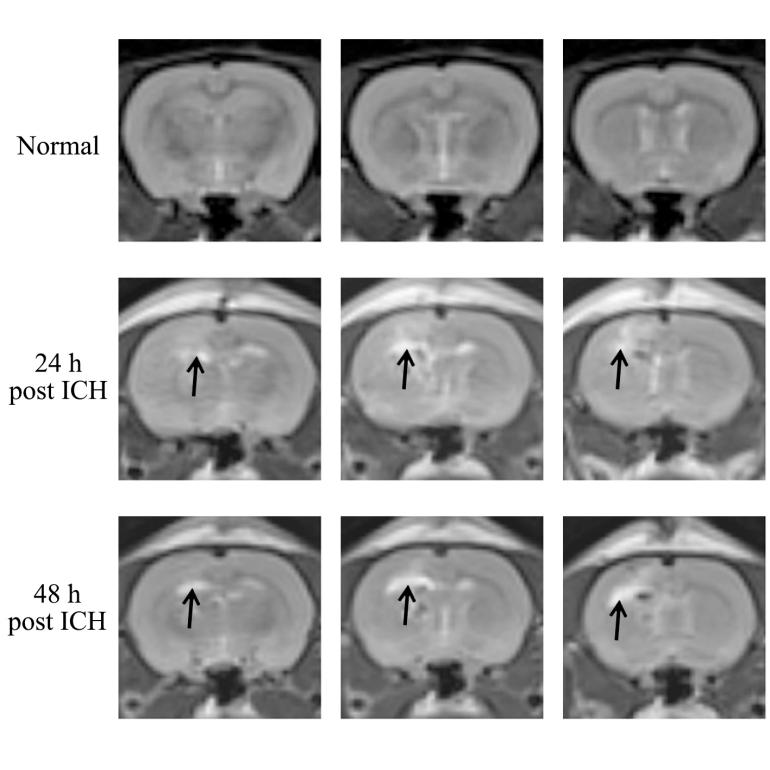
**

Supplement Fig 1. The T2-weighted imaging results of head scanning in SD rats were obtained using a 3.0T Siemens NMR device. The figure displays brain MRI images of normal rats, as well as those taken at 24 hours (24 h post ICH) and 48 hours (48 h post ICH) after cerebral hemorrhage occurrence. In the brain MRI of normal rats, no low signal area indicative of bleeding was observed. However, in the images captured at 24h post ICH and 48h post ICH, marked by the black arrow, a highlighted low signal was evident in T2-weighted imaging.

**Supplement Figure 2**


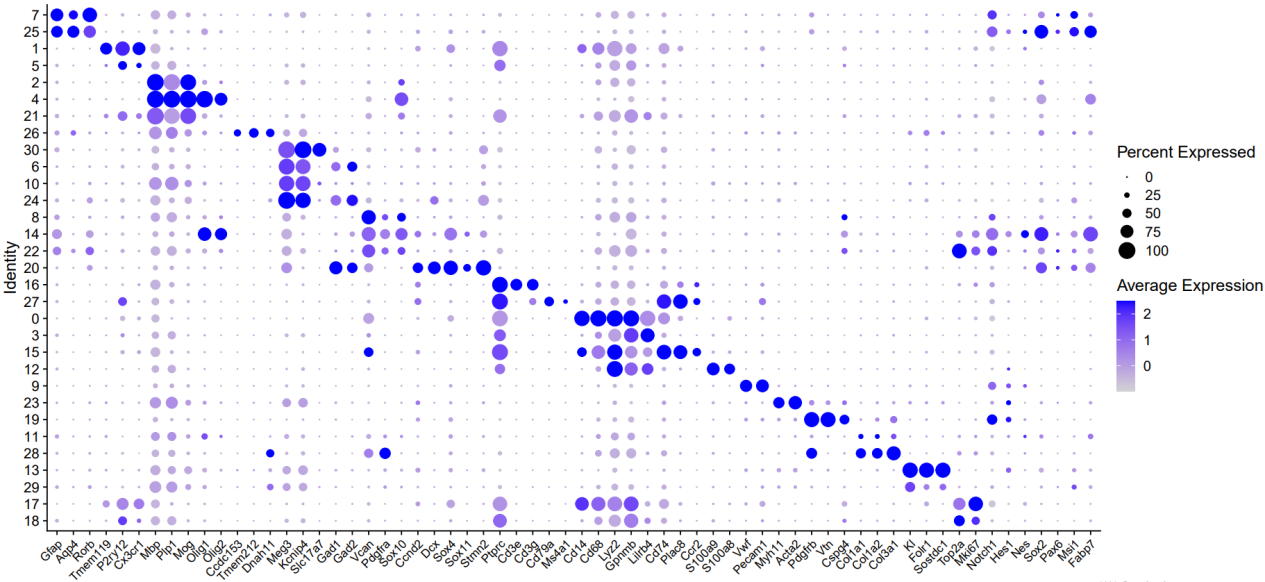


Supplement Fig 2. Dot plots of representative genes specific for the indicated cell subtypes. The size of each dot represents the cell percentage of this population positive for the marker gene. The scale of the dot color represents the average expression level of the marker gene in this population.

**Supplement Figure 3**


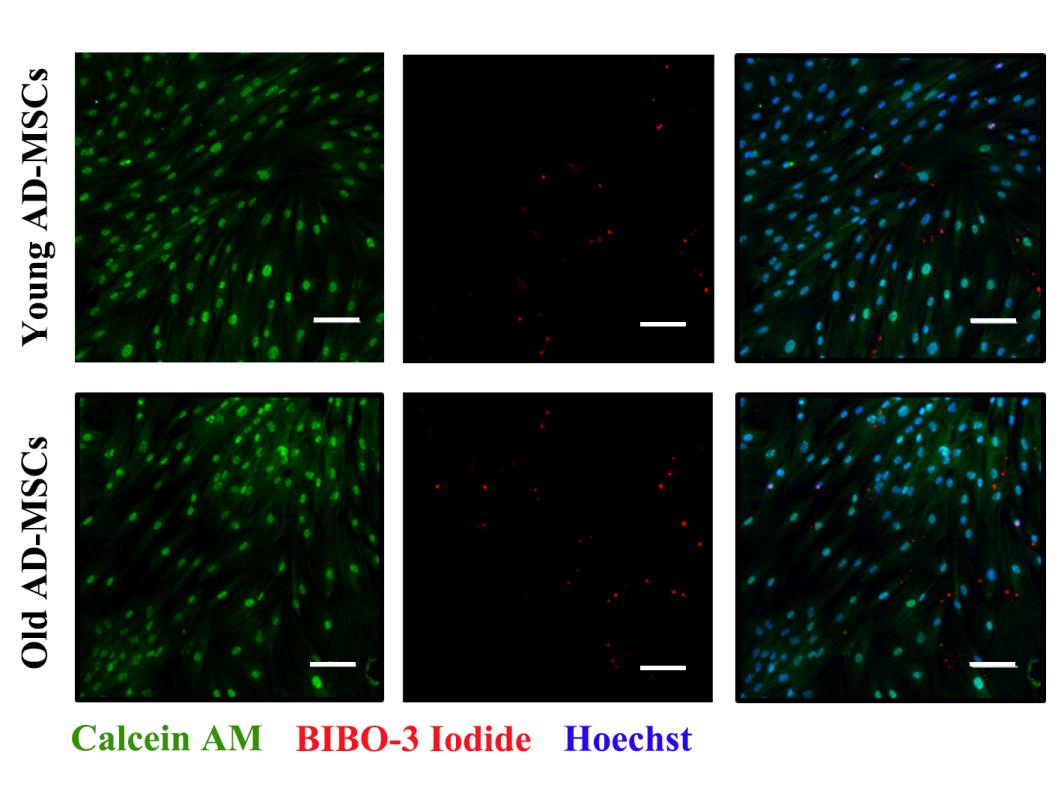


Supplement Fig 2. The viability test results of AD-MSCs by LIVE/DEADTM cell imaging kit (Cat No. R37601, Thermo Fisher Scientific, USA) and NucBlueTM live cell stain. Viable cells are stained with green fluorescence; the nuclei of viable cells are stained with blue fluorescence; dead cells are stained with red fluorescence. Bar=100 μm.
